# Complement factor I deficiency: A potentially treatable cause of fulminant cerebral inflammation

**DOI:** 10.1212/NXI.0000000000000689

**Published:** 2020-02-25

**Authors:** Tom Altmann, Megan Torvell, Stephen Owens, Dipayan Mitra, Neil S. Sheerin, B. Paul Morgan, David Kavanagh, Rob Forsyth

**Affiliations:** From the Department of Paediatric Infectious Disease (T.A, S.O.), Newcastle upon Tyne Hospitals NHS Foundation Trust; Division of Infection and Immunity and Dementia Research Institute (M.T., B.P.M.), School of Medicine, Cardiff University; Department of Neuroradiology (D.M.), Newcastle upon Tyne Hospitals NHS Foundation Trust; Department of National Renal Complement Therapeutics Centre (N.S.S., D.K.), Newcastle upon Tyne Hospitals NHS Foundation Trust; Complement Therapeutics Research (N.S.S., D.K.), Translational and Clinical Research Institute, Newcastle University; Department of Paediatric Neurology (R.F.), Newcastle upon Tyne Hospitals NHS Foundation Trust; Neuroscience, Neurodisability and Neurological Disorders Groups (R.F.), Translational and Clinical Research Institute, Newcastle University, United Kingdom.

## Abstract

**Objective:**

To raise awareness of complement factor I (CFI) deficiency as a potentially treatable cause of severe cerebral inflammation.

**Methods:**

Case report with neuroradiology, neuropathology, and functional data describing the mutation with review of literature.

**Results:**

We present a case of acute, fulminant, destructive cerebral edema in a previously well 11-year-old, demonstrating massive activation of complement pathways on neuropathology and compound heterozygote status for 2 pathogenic mutations in CFI which result in normal levels but completely abrogate function.

**Conclusions:**

Our case adds to a very small number of extant reports of this phenomenon associated with a spectrum of inflammatory histopathologies including hemorrhagic leukoencephalopathy and clinical presentations resembling severe acute disseminated encephalomyelitis. CFI deficiency can result in uncontrolled activation of the complement pathways in the brain resulting in devastating cerebral inflammation. The deficit is latent, but the catastrophic dysregulation of the complement system may be the result of a C3 acute phase response. Diagnoses to date have been retrospective. Diagnosis requires a high index of suspicion and clinician awareness of the limitations of first-line clinical tests of complement activity and activation. Simple measurement of circulating CFI levels, as here, may fail to diagnose functional deficiency with absent CFI activity. These diagnostic challenges may mean that the CFI deficiency is being systematically under-recognized as a cause of fulminant cerebral inflammation. Complement inhibitory therapies (such as eculizumab) offer new potential treatment, underlining the importance of prompt recognition, and real-time whole exome sequencing may play an important future role.

We report a case of life-threatening, nonhemorrhagic fulminant CNS inflammation, radiologically resembling acute disseminated encephalomyelitis (ADEM), in association with complete complement factor I (CFI) functional deficiency. A very few such cases have been reported to date, all identified retrospectively via whole exome sequencing (WES) and/or known family history. Complement inhibition (e.g., with eculizumab) represents a potential therapeutic option in this otherwise devastating illness but would require prompt recognition. The index case had a functional CFI deficiency (with *normal* serum CFI levels), emphasizing that simple serum complement assays will not exclude CFI deficiency and the challenge of timely diagnosis. CFI deficiency may be an under-recognized cause of encephalitis of “presumed viral” or unknown etiology. Improved outcome will require greater awareness of the condition and a high index of suspicion.

## Case

An 11-year-old Caucasian girl presented with a 5 days history of fever, headache, and vomiting. She had no significant medical history and no recent foreign travel. Initial GCS was 14 of 15 but rapidly fell to 8. She was intubated and ventilated. Temperature on admission was 37.6°C. White blood cell count was 14.3 mm^−3^, with 90% neutrophils with an erythrocyte sedimentation rate of 103 mm^—^/h. C-reactive protein was 201 mg/L.

**Figure 1:**
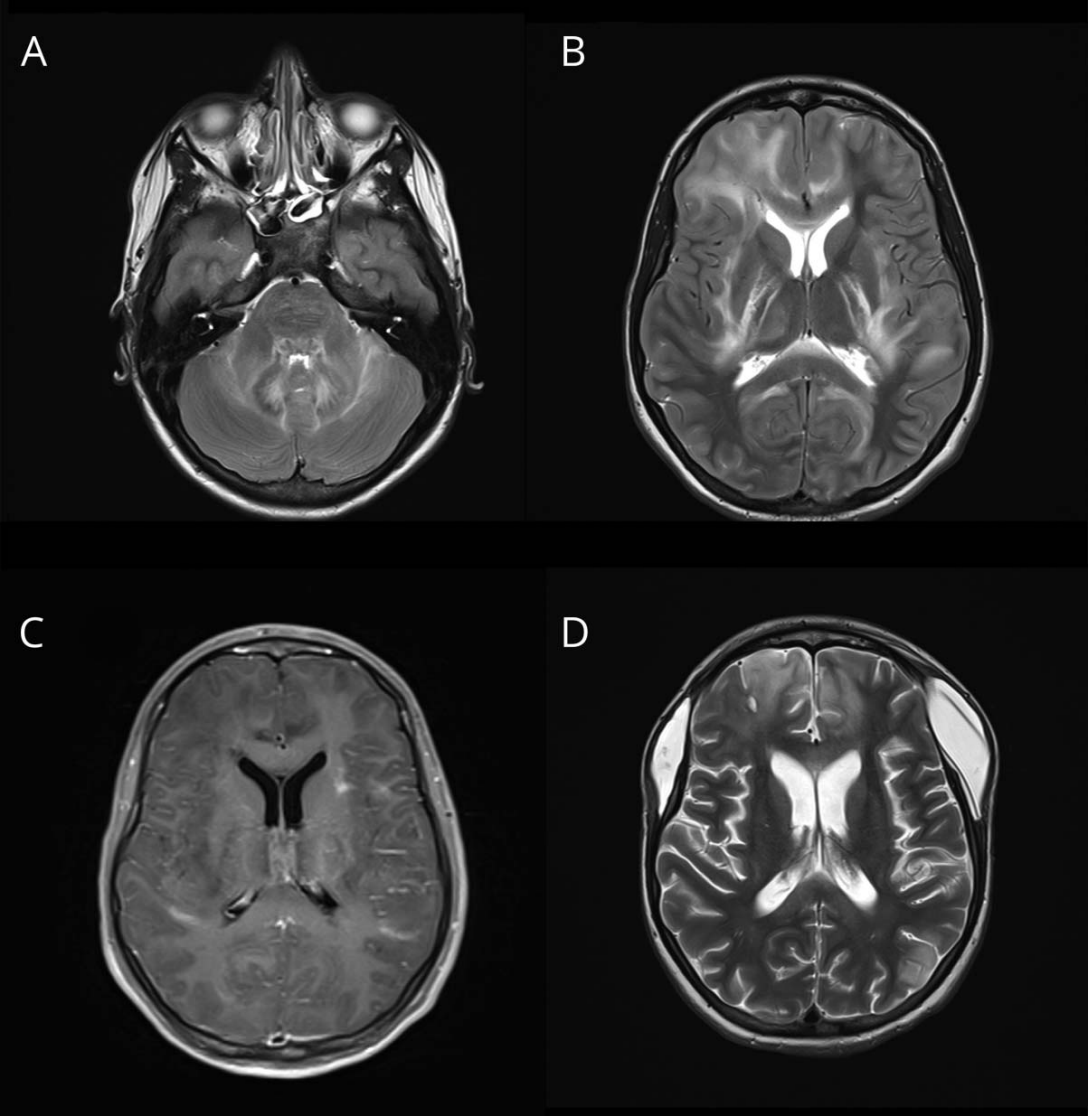
Representative MR images Representative neuroradiologic images. (A–C) Acute imaging on day 2 of admission shows bilateral, asymmetrical, predominantly white matter changes, although some gray matter involvement of thalami is also seen. Patchy enhancement postcontrast and mass effect and effacement of the sulci. Diffusion-weighted imaging (not shown) did not indicate any area of restricted diffusion. (D) Approximately 1 month later showing postcraniectomy changes and substantial resolution of the acute inflammation. (A, B, and D = T2-weighted; C = postcontrast T1-weighted).

**Figure 2:**
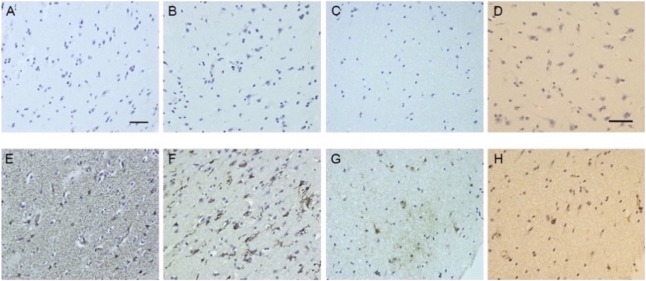
Immunohistochemistry Immunohistochemistry of parietal cortical sample obtained at the time of craniectomy demonstrate reactive astrogliosis, microgliosis, and complement deposition. Top row (A–D) indicates controls (secondary antibody only). Bottom row (E–H) indicates antibody staining. A and E, Reactive astrocyte marker glial fibrillary acidic protein. B and F, Pan-microglial marker ionized calcium binding adapter molecule 1. C and G, In-house anti-C3b/iC3b monoclonal antibody C3/30. D and H, Anti-C9 neoantigen-specific monoclonal antibody B7 (membrane attack complex). Scale bars = 50 μm.

Initial CT of the head showed no bleed or mass. Initial MRI (figure 1) demonstrated bilateral, asymmetrical, predominantly white matter edema with posterior corpus callosal changes; some gray matter involvement of thalami; and patchy enhancement postcontrast. There was no restricted diffusion. She developed rapidly progressive, life-threatening cerebral edema requiring an external ventricular drain followed by bifrontal decompressive craniectomy at which point a superficial cortical brain biopsy was obtained. There were no significant light microscopic abnormalities. Immunohistochemical studies showed no evidence of a demyelinating process with few T cells in the tissue. There was marked astrogliosis (indicated by glial fibrillary acidic protein staining, figure 2E) and microgliosis (ionized calcium binding adapter molecule 1 staining, figure 2F) accompanied by deposition of C3b/iC3b (figure 2G) and terminal complement complex (figure 2F), both of which appear neuronal in location.

She was treated with ceftriaxone and aciclovir for presumed meningoencephalitis, high-dose methylprednisolone (1 g daily for 5 days), followed by an extended high-dose enteral prednisolone taper; plasmapheresis with human albumin solution and fresh frozen plasma (FFP) (days 5–15 inclusive); and rituximab (total 1125 mg/m^2^ in 2 doses, days 6 and 21, because of concern about possible washout of the first dose with plasmapheresis) for a working diagnosis of severe ADEM. There was little obvious benefit. The severely elevated intracranial pressure began to settle around day 6. By day 14, some withdrawal from painful stimuli was noted. Blood and CSF cultures were negative for primary bacterial infection. CSF virology was negative for herpes simplex, varicella, *Enterovirus*, and *Parechovirus* by PCR. An initial CSF sample was heavily blood-stained with protein 0.81g/L and 30,000 erythrocytes/mm^3^ but no excess of CSF leukocytes. Subsequent CSF samples showed no CSF leukocytes. Antibodies to myelin-oligodendrocyte glycoprotein, aquaporin 4, glycine receptors, voltage-gated potassium channel-related proteins, and NMDA receptors were all negative. Immunoglobulin levels at presentation were normal.

Retrospectively, a neuroinflammatory gene panel identified 2 pathogenic CFI variants (c.1019T>C p.(Ile340Thr) and c.1555 G>A p.(Asp519Asn). Subsequent Sanger sequencing of the proband and parents confirmed compound heterozygote status. Subsequent complement assays were performed 8 months after presentation. C3 and C4 were within the normal range (0.72 [normal range (NR) 0.68–1.80] and 0.35 [NR 0.18–0.6] g/L, respectively). CFI and complement factor H (CFH) were within the normal range (29 mg/L [NR 21–40] and 0.59 [NR 0.56–0.94] g/L, respectively). There was marked activation of the alternative pathway (AP) with markedly low complement factor B (CFB; 49 mg/L: NR > 186 mg/L) and absent AP haemolytic activity (AP100).

Nearly 2 years from presentation, the patient has been left with a profound neurologic deficit with tetraplegia, although she can write slowly with specialist aids indicating relative cognitive preservation. A seizure disorder is well controlled with levetiracetam. She has commenced penicillin prophylaxis and has been immunized against capsulated organisms in light of the absent AP activity. Follow-up imaging at 6 months showed severe widespread atrophy with no new signal abnormality (supplemental data figure 2, links.lww.com/NXI/A208).

## Discussion

**Figure 3:**
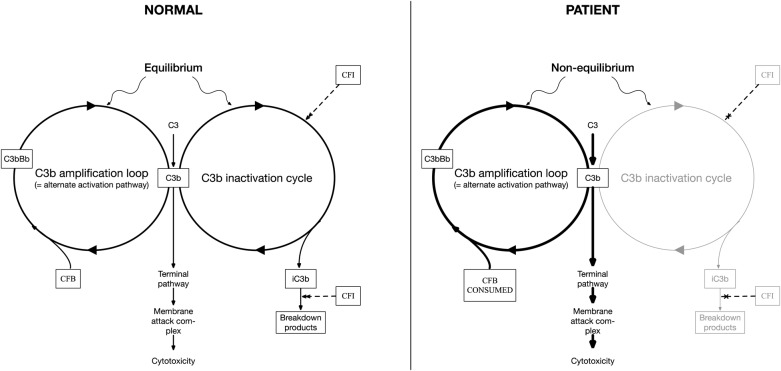
Complement biology Highly simplified cartoon of complement amplification loop pathways indicating (left, normal healthy condition) how C3/C3b levels reflect an equilibrium between 2 cycles: one of C3 cleavage to form C3b that interacts with factor B, which is then cleaved by factor D to form the C3 convertase (C3bBb) to cleave more C3 and a CFI-dependent C3b inactivation cycle. In factor I deficiency (right), a lack of C3b inactivation causes uncontrolled accumulation of C3b, rapid consumption of C3, and activation of the downstream terminal pathway. Excessive activation of the C3b amplification loop can be inferred from CFB levels that are markedly reduced because of consumption. CFB = complement factor B; CFI = complement factor I.

We describe a patient with compound heterozygote, complete CFI deficiency, with a nonclassic, neurologic presentation. CFI is a complement regulatory protein that inactivates all pathways of the complement system by cleaving the α′-chains of C3b and C4b (figure 3). Complete CFI deficiency is believed to be rare and, as would be predicted, has mainly been reported in association with primary infection (including CNS infection) with encapsulated microorganisms.^1^ Heterozygote status for CFI deficiency has been associated with atypical hemolytic uraemic syndrome (aHUS) (i.e., not associated with *Escherichia coli* gastroenteritis)^2^ and age-related macular degeneration.^3^ CFI-mediated complement regulation may also be important in the progression of early phase Alzheimer disease.^4^

A report of 2 unrelated children of Filipino descent^5^ and a recent adult brief report^6^ describe acute hemorrhagic leukoencephalopathy (AHLE) in association with complete CFI deficiency. All had infrequently recurrent episodes, at intervals of several years, commencing in the second decade of life. Radiologically, our case resembles case B in Shields et al.*,*^6^ but appears uniquely severely affected among cases reported to date.

We speculate that the basis for such infrequent, fulminant presentations may be that the CFI deficiency causes chronic AP activation limited only by depletion of its substrate C3. C3 is an acute phase protein. and stressors such as viral infection may result in a rapid surge in C3 production, freeing the previously substrate-limited AP and resulting in a catastrophic runaway that overwhelms downstream complement regulators (which are in any case poorly expressed in the brain). In this context, one could hypothesize that our use of plasmapheresis with FFP may have been deleterious because of delivery of exogenous (functional) factor I and C3, facilitating proinflammatory conversion of C3b to iC3b (figure 3), although no clinical deterioration was detectable at the time. We were not able to identify an infective trigger in our case although she had been pyrexial before admission.

Our case had no radiologic or pathologic evidence of hemorrhage, suggesting an AHLE phenotype is not consistent and that neuroradiologic appearances can resemble acute demyelination. Complement activation is a recognized pathophysiologic mechanism in neuromyelitis optica and related disorders,^7,8^ suggesting a basis for similar radiologic appearances.

Improved outcomes are likely to depend on prompt recognition of the CFI deficiency to administer anticomplement drugs. This case highlights several diagnostic pitfalls. The patient's I340T and D519N variants have been reported (as heterozygotes) in association with aHUS (see supplemental data, links.lww.com/NXI/A208). In both instances, absolute levels of CFI were normal; however, functional analysis demonstrated almost complete abrogation of enzymatic activity.^9^ Thus, unlike previous cases,^5^ our patient was in the highly unusual situation of having antigenically normal CFI levels but severely deficient CFI activity from both mutant alleles. In addition, the unexpected finding of normal serum C3 level in the serum reflects the fact that routine clinical assays may also detect other forms of C3, in this setting, most likely C3b. The most reliable routinely clinically available diagnostic clue in this situation is the markedly reduced CFB level that reflects excessive CFB consumption because of a lack of inhibition of the amplification loop (figure 3).

In keeping with other reports,^5^ our patient showed no response to plasma exchange or rituximab, although case B in Shields et al.^6^ did show a response to methylprednisolone. Broderick et al.^5^ saw modest benefits coinciding with anakinra use. Eculizumab is the only currently available complement inhibitor. It is a monoclonal antibody that specifically binds to C5 and blocks its cleavage, preventing formation of the membrane attack complex and C5a release. There is growing interest in the potential of eculizumab in severe autoimmune demyelinating neuroinflammatory disease, particularly anti-aquaporin 4-mediated neuromyelitis optica.^8^ In future cases of unexplained ADEM or AHLE, we suggest that clinicians investigate the presence of a potential complement regulation defect via extended complement assays (including complement function, activation products, and levels of CFI, CFH, and CFB). Real-time WES^10^ may also play an important future role in diagnosis. The identification of complement regulator defects would provide a biological rational for the use of eculizumab and the next wave of 2nd generation complement inhibitory therapies.

Deidentified clinical data will be made available on appropriate request to the corresponding author.

## Glossary

ADEM: acute disseminated encephalomyelitis
AHLE: acute hemorrhagic leukoencephalopathy
aHUS: atypical haemolytic uraemic syndrome
AP: alternative pathway
CFB: complement factor B
CFH: complement factor H
CFI: complement factor I
FFp: fresh frozen plasma
WES: whole exome sequencing
